# Annotation of *Fusarium graminearum* (PH-1) Version 5.0

**DOI:** 10.1128/genomeA.01479-16

**Published:** 2017-01-12

**Authors:** Robert King, Martin Urban, Kim E. Hammond-Kosack

**Affiliations:** Rothamsted Research, Harpenden, Herts, United Kingdom

## Abstract

*Fusarium graminearum* floral infections are a major risk to the global supply of safe cereal grains. We report updates to the PH-1 reference genome and significant improvements to the annotation. Changes include introduction of legacy annotation identifiers, new gene models, secretome and effectorP predictions, and inclusion of extensive untranslated region (UTR) annotations.

## GENOME ANNOUNCEMENT

The ascomycete fungus* Fusarium graminearum* is one of several commonly found pathogenic species identified on flowering wheat spikes that cause yield losses, grain quality reductions, and significant mycotoxin contamination in the United Kingdom, Europe, the Americas, Asia, and elsewhere. In 2014, the reference genome for isolate PH-1 was nearly completed with just two remaining gaps (1). In this study the remaining sequence gaps and misassemblies were corrected using new assembler software and the annotation updated. In the United Kingdom, a second pathogen of major concern is *Fusarium culmorum* for which a fragmented genomic reference sequence exists for the United Kingdom isolate UK99 (2). Both species are closely related and produce a range of mycotoxins including the type B trichothecene deoxynivalenol and its derivatives (3). Harvested grains contaminated with trichothecene mycotoxins negatively affect farmers profit margins and impact upon global food and feed security.

Here we report further updates for the reference genome PH-1 of *Fusarium graminearum*. The two remaining sequence gaps were closed by mapping previously not aligned PH-1 reads to the closed gaps in other closely related *F. graminearum* isolates (unpublished genomes). An extensive update of the annotation (14,145 genes) has been included to adopt a new gene naming schema, include legacy annotation gene ID’s, and to re-annotate genes with a focus upon those lacking an ATG as a start codon. A new prediction of the secretome has resulted in a reduced cohort of gene predictions (*n* = 870), much more in line with those recently identified in other fungal pathogens species, of which 97 are predicted to be GPI-anchored. We also used effectorP to predict a sub-set of secreted effector proteins (*n* = 183), and updated the gene models with 5,694 5′ and 5,036 3′ untranslated region (UTR) annotations.

The *F. graminearum* strain PH-1 annotation was transferred to the new assembly via masker 2 (4) followed by a re-annotation using AUGUSTUS (5), and GeneMark (6) to update gene models. Curation was performed on the pre-identified genes lacking an ATG start codon using Geneious (version 9.1 created by Biomatters) as the browser with the new gene models. Secretome predictions were done by the identification of proteins with a signal peptide, but lacking a transmembrane domain. Loci coding for extracellular proteins were predicted using WoLF PSORT (7), followed by Big-PI to predict GPI-anchors (8). The subset of fungal effector proteins was further characterized using EffectorP (9).

### Accession number(s).

Raw data and the assembled sequences have been updated in the European Nucleotide Archive (ENA). The study accession number is PRJEB5475. Accession numbers for the assembled chromosomes are HG970330 to HG970335. Secretome and effector predictions can be found at https://goo.gl/PKlnwu.
